# Deep Denoising of Wavefront Sensor Signals via Physics-Aware Dual-Channel Decoupled Network (PRISM)

**DOI:** 10.3390/s26123831

**Published:** 2026-06-16

**Authors:** Jianbao Ma, Yun Pan, Yiyou Fan, Hao Wang, Jinshan Su

**Affiliations:** Key Laboratory of Signal Intelligent Capture and New Generation Communication Technology, School of Electronic Engineering, Yili Normal University, 448 Jiefang Road, Yining 835000, China; 20240050184@ylnu.edu.cn (J.M.); 20230050200@ylnu.edu.cn (Y.P.); 14012@ylnu.edu.cn (Y.F.); 20250050227@ylnu.edu.cn (H.W.)

**Keywords:** signal enhancement, deep learning, submillimeter-scale vibration signals, wavefront sensors

## Abstract

Laser remote sensing based on wavefront sensors shows great potential for detecting minute vibrations. However, due to their high detection sensitivity, wavefront sensors are highly susceptible to interference from environmental noise and instrument-induced noise, which significantly compromises the quality of the acquired vibration signals and the accuracy of the detection. In this study, over 60,000 vibration signal data samples were collected under various amplitude and frequency conditions using a laser remote sensing seismic wave detection system. By applying a physics-aware dual-channel decoupled network (PRISM) to perform noise reduction on the vibration signals, we achieved improvements in signal quality under multiple real-world noise environments. The average signal-to-noise ratio improved by 12.16 dB, and the signal distortion ratio improved by 6.35 dB, successfully preserving faint vibration signals within the noise.

## 1. Introduction

Laser-based seismic wave monitoring and precision microseismic measurement technologies are advancing rapidly, offering new approaches to geological research and deep resource exploration [1,2,3,4]. Compared to the bulky and complex optical paths of traditional interferometers, wavefront sensors [5,6], which require no physical reference arms, feature compact systems and offer high-spatial-resolution array detection capabilities, are finding widespread application in complex field geological exploration and long-distance microseismic measurement.

The wavefront sensor uses a micro-lens array to map the physical phase distortion of the laser light reflected by the target into geometric displacement of the spot on the focal plane, thereby enabling precise decoding of microscale changes in the target. Wavefront sensors not only enable high-sensitivity measurements at the submillimeter level, but they also simultaneously capture dynamic deformation characteristics at multiple points in real time. This high-precision data acquisition capability effectively overcomes the low signal acquisition efficiency of traditional seismic remote sensing methods in complex environments, making it an effective method for capturing faint surface vibration signals.

However, the practical application of wavefront sensors in complex engineering environments is often subject to noise interference from various external physical factors. The primary sources of noise include random wind disturbances, sunlight, and random environmental vibrations. In addition, noise such as photon scattering and CMOS readout interference may also exist within the optical system. This complex combination of interference results in raw signals with an extremely low signal-to-noise ratio, causing the extremely fragile sub-millimeter-scale peaks of real targets to be completely overwhelmed, thereby significantly reducing the physical fidelity of the signal and subsequent detection accuracy.

At the same time, work environments such as complex field geological exploration and long-distance microseismic surveys are typically unpredictable. Although signals acquired in these environments generally exhibit low signal-to-noise ratios, the specific S/N ratios can vary due to numerous environmental noise factors, including terrain and climate conditions. This makes it impossible for traditional filtering methods to effectively process data under such complex conditions.

Traditional signal enhancement methods, such as Wiener filtering, wavelet transform, and empirical mode decomposition, often yield limited results and generally perform poorly when processing signals with low signal-to-noise ratios in complex operational environments. This is because these traditional methods typically rely on prior knowledge and experience to analyze noise characteristics before performing signal enhancement, which makes it difficult to apply them to various real-world scenarios under complex operating conditions.

Although deep learning has achieved significant success in speech enhancement and image restoration [7,8,9], applying mainstream architectures directly to wavefront sensor signals with extremely low signal-to-noise ratios (SNR) reveals fundamental limitations. Traditional real-domain spatio-temporal networks (such as U-Net and its variants [10,11,12]) focus on macroscopic energy mapping, making them prone to misclassifying weak high-frequency transient harmonics as noise, resulting in severe “oversmoothing” and the loss of critical physical peaks. One-dimensional end-to-end temporal networks (such as Conv-TasNet [13,14]) lack the spatial receptive fields necessary to capture two-dimensional optical phase continuity, making it difficult to track transient Doppler shifts. Even state-of-the-art complex neural networks (such as DCCRN [15,16]) suffer from severe “phase noise cross-coupling” under strong noise conditions, where their strict complex multiplication rules actually amplify phase distortions. These limitations starkly highlight the absence of physical awareness mechanisms in existing models, creating an urgent need for a novel network architecture tailored specifically for the recovery of weak wavefront signals.

To preserve more phase information during the noise reduction process and improve the noise reduction performance of vibration signals, this study collected over 60,000 vibration signal data samples over nearly three months using a laser remote sensing detection system based on wavefront sensors. A neural network architecture for vibration signal noise reduction—PRISM (Physics-Aware Dual-Channel Decoupled Network)—was designed and applied, achieving effective noise reduction in vibration signals. PRISM introduces a “2+1”-dimensional physics-enhanced tensor input and utilizes amplitude envelopes to guide the network in precisely refining phase information; To address the challenge of easily lost sub-millimeter-scale vibrations, a “coarse-to-fine” cascaded architecture combining CBAM (Convolutional Block Attention Module) with spatiotemporal attention was designed to better preserve microscopic features; a physical joint loss term incorporating log-spectral distance was also proposed, which utilizes gradient amplification to resolve the issue of excessive noise suppression.

The results demonstrate that the PRISM neural network architecture can effectively reduce noise in sub-millimeter-level vibration signals under real laboratory conditions and with varying synthetic signal-to-noise ratios. It preserves weak vibration signals and original features within the noise, and all evaluation metrics show improvements compared to the pre-processing stage. This lays the foundation for the future processing of vibration signals collected under various complex operating conditions.

## 2. Laser Remote Sensing Detection Systems and Dataset Construction

### 2.1. Working Principle of the Wavefront Sensor

Seismic waves can be classified into three types based on their mode of propagation: P-waves (primary waves), S-waves (secondary waves), and surface waves. Among these, S-waves can only propagate through solids, causing horizontal ground motion, and are relatively difficult to detect, while surface waves are mostly interference waves. P-waves, on the other hand, are progressive waves and are the first seismic waves to reach the Earth’s surface; not only do they exhibit distinct observational characteristics, but they are also the primary target of seismic detectors.

**Figure 1:**
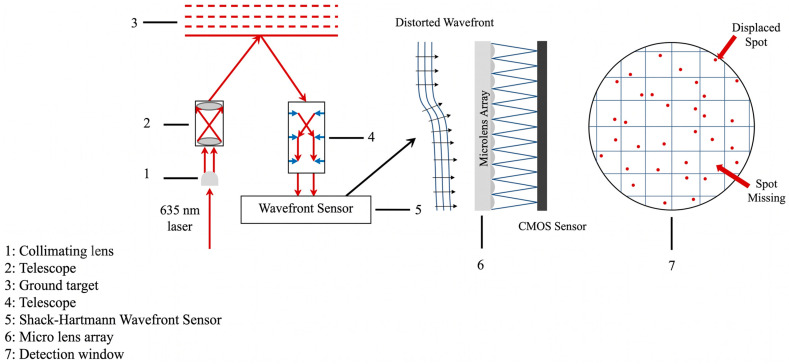
Vibration signal detection system.

The detection system used in this paper is physically divided into two main modules: the transmitter and the receiver. The transmitter integrates a laser, fiber collimation components, and a telescope to stably output a continuous-wave laser beam at 635 nm with constant power. Beam shaping ensures that the projected light field exhibits strict spatial uniformity. The receiver collects target echoes through the full aperture of the telescope, and the beam is directly projected onto the core component, the wavefront sensor. The wavefront sensor utilizes a micro-lens array (MLA) to achieve independent sampling across multiple spatial channels. The lenses have an effective aperture of 146 μm and a focal length of 4 mm, and they are coupled to a CMOS imaging sensor (software version: 18183-D03). In terms of the detection mechanism, the MLA performs two-dimensional spatial discretization of the incident wavefront, independently focusing sub-aperture beams onto detection windows, thereby providing a high-resolution spot image source for subsequent wavefront slope signal extraction. The detection system is shown in Figure 1.

**Figure 2:**
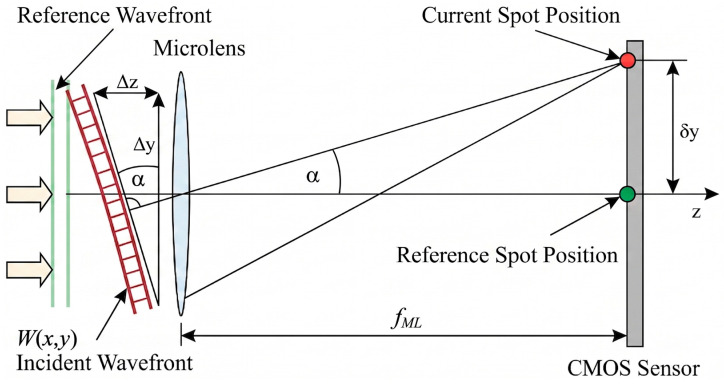
Physical mechanism of wavefront distortion in wavefront sensor microlenses.

When the target surface (such as the ground excited by seismic P-waves) undergoes slight vibrations, the reflection or scattering angle of the incident detection beam changes dynamically, thereby introducing spatial phase distortion into the returning laser wavefront. Figure 2 visually illustrates the core physical mechanism through which individual microlenses within a Shack-Hartmann wavefront sensor capture and quantify such phase distortion.

When an undisturbed flat wavefront (shown as the green double solid line in the figure) enters the microlens perpendicularly, the beam is precisely focused at the center of the lens’s optical axis, forming a reference spot (green dot) on the CMOS sensor surface. When a distorted wavefront (Incident Wavefront *W*(*x,y*), shown as the red zigzag line in the figure), carrying information about minute surface vibrations, passes through the effective subaperture of the microlens, the direction of the collected light beam is deflected due to the local inclination angle *α* of the wavefront. This causes the focal point on the CMOS focal plane to deviate from its original central position, forming the current measurement spot (red dot).

This spatial distortion of the wavefront phase is ingeniously converted into a physical lateral displacement of the spot array on the focal plane. The displacement δy of the center of mass of the measured spot relative to the reference spot has a strict geometric mapping relationship to the intrinsic focal length *f_ML_* of the microlens and the local slope (inclination angle *α*) of the wavefront. By comparing the positional changes δy between the measured spot and the reference spot in real time, the wavefront slope information reflecting the dynamic displacement of the ground surface can be accurately calculated.

To further quantify the deterministic relationship between the wavefront slope and the actual physical vibration amplitude (Δ*z*) at the surface, this section establishes a rigorous mathematical model based on geometric optics. According to the geometric imaging principle shown in Figure 2, the focal length *f_ML_* of the microlens, the wavefront inclination angle *α*, and the offset Δ*s* of the spot center satisfy the following strict trigonometric relationship:

(1) $$\tan(\alpha )=\frac{\Delta s}{f_{ML}}$$

Given that the phase distortion of the wavefront caused by surface submillimeter microseismic excitation is extremely weak, the local dip angle typically satisfies the condition of being extremely small (α ≪ 1). Therefore, using the first-order small-angle approximation (tan(α)≈α), the above geometric relationship can be linearly simplified to:

(2) $$\alpha \approx \frac{\Delta s}{f_{ML}}$$

**Figure 3:**
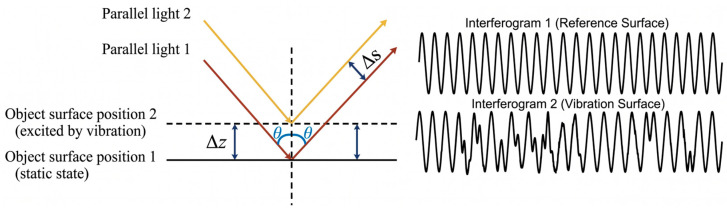
Relationship between the target surface and the reflected laser beam.

To establish a relationship between this wavefront tilt angle *α* and the actual physical vibration amplitude Δ*z* at the ground surface, it is necessary to further quantitatively analyze the evolution of the reflected probe laser. Assume that a collimated probe laser beam illuminates the target area at an angle of incidence *θ* relative to the normal of the target surface (as shown in Figure 3). When the surface is excited by a longitudinal wave and undergoes a transient vertical displacement Δ*z*, the physical reflection surface of the laser will undergo a corresponding parallel translation. Geometrically, this vertical translation causes a lateral displacement Δd in the reflected echo’s optical path in space, and its projection relationship can be expressed as:

(3) Δd=Δz·sin(2θ)

Subsequently, the reflected beam, which has undergone a lateral displacement of Δd, is collected by the receiving telescope system. Let the lateral magnification of the telescope system be *M*; then, the spatial displacement of the beam as it is projected onto the micro-lens array (MLA) plane is modulated to Δx=M·Δd. At the same time, since the echo scattered from the target surface possesses an inherent wavefront curvature (let the radius of curvature of the wavefront at the MLA receiving plane be *R*), the spatial displacement Δx of the beam forces the fixed micro-lenses to sample different local regions of the wavefront. The resulting local change in wavefront slope, Δα, is linearly spatially coupled with the spatial displacement, i.e., $\Delta \alpha =\frac{\Delta x}{R}$. Substituting the displacement formula into this coupling relationship yields the functional equation relating the change in wavefront slope to the physical amplitude of the ground surface:

(4) $$\Delta \alpha =\frac{M\cdot \sin(2\theta )}{R}\cdot \Delta z$$

Since the change in local wavefront slope Δα caused by the translation of the optical path is physically equivalent to the actual angular deflection (i.e., Δα=α) at the entrance of the microlens sub-aperture, the complete cross-domain optical-mechanical geometric mapping equation can be derived by solving the system of the above derived equations:

(5) $$\frac{\Delta s}{f_{ML}}=\frac{M\cdot \sin(2\theta )}{R}\cdot \Delta z$$

By rearranging the terms and performing algebraic manipulation on the above equation, we can ultimately establish a strict linear proportional relationship Δz=K·Δs between the amplitude Δ*z* of the transient surface vibration and the displacement Δ*s* of the spot’s center of mass, where the analytical expression for the conversion ratio coefficient *K* is strictly defined as:

(6) $$K=\frac{R}{M\cdot f_{ML}\cdot \sin(2\theta )}$$

In this system of physical equations, the proportionality constant *K* serves as an intrinsic “conversion factor” for the system, profoundly revealing the transmission gain involved in the evolution of microscopic optical phase characteristics into macroscopic mechanical vibration characteristics. Its physical nature is jointly determined by four core boundary conditions: the focal length *f_ML_* determines the angular amplification of phase distortion by the microlens sub-aperture; the angle of incidence θ determines the geometric projection efficiency of the conversion from vertical vibrational displacement to lateral beam shift; the magnification factor *M* determines the scaling factor of the spatial field of view for the optical receiving antenna; and the radius of curvature *R* characterizes the inherent degree of space-phase coupling in the reflected light field. The comprehensive mathematical derivation established based on this scaling factor not only rigorously demonstrates the scientific validity of the linear proportionality of Δz∝Δs from fundamental optical principles but also provides a solid theoretical foundation for the subsequent PRISM network to accurately learn the noise-contaminated two-dimensional wavefront slope characteristics, thereby achieving high-fidelity physical reconstruction of submillimeter-scale surface microseismic signals.

### 2.2. The Relationship Between Wavefront Slope and Amplitude and the Necessity of Noise Reduction

**Figure 4:**
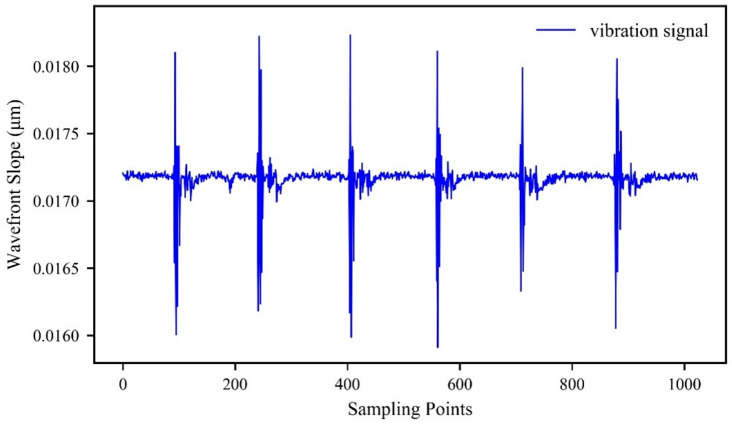
Wavefront slope signals recorded during vibration.

The propagation of longitudinal waves (such as seismic waves) causes slight vertical vibrations at the Earth’s surface, which can be intuitively interpreted as changes in the physical amplitude of the ground. In practical detection, a controlled source is used to excite the target surface with varying frequencies and intensities, and the receiving end of a Shack-Hartmann wavefront sensor is aligned vertically with the target surface to simulate and capture the effects of longitudinal waves on the laser wavefront. When the probe laser is reflected back from the excited ground, the instantaneous changes in the ground surface position cause corresponding phase distortions in the laser wavefront, thereby embedding information about the ground’s dynamic vibrations within the echo signal. The relationship between the target surface and the reflected laser is shown in Figure 3, and the wavefront slope signal acquired during vibration is shown in Figure 4.

Due to wavefront distortion, when the micro-lens array inside the sensor performs discrete focusing, it causes a dynamic deviation in the position of the spot formed on the CMOS pixel plane. Theoretical and experimental results confirm that the displacement of the spot’s center of mass (Δ*s*) specifically carries information about the target’s vibration and exhibits a strictly linear relationship with the instantaneous physical amplitude (Δ*z*) of the ground surface (Δz∝Δs) [17]. Normalizing the horizontal and vertical center-of-mass displacements by the microlens focal length converts them into the wavefront slope at that measurement point. Through an inverse integration on the two-dimensional wavefront slope matrix (similar to solving the Poisson equation), the wavefront phase can be accurately reconstructed. Subsequently, utilizing the linear relationship between phase and amplitude, sub-millimeter-scale micro-excitation amplitudes can be calculated.

However, in real-world physical environments, phase reconstruction based directly on the raw wavefront slope often presents significant challenges. When detecting submillimeter-scale, faint vibrations at extremely low signal-to-noise ratios, the laser echo signal is extremely weak and highly susceptible to various physical interferences, both from within the sensor and from the external environment. Therefore, high-precision intelligent noise reduction and interference suppression of the wavefront slope signal are essential.

### 2.3. Environmental Noise Analysis

In an ideal laboratory environment, wavefront slope signals clearly reflect the vibrations of the target. However, in real-world long-range laser remote sensing and complex engineering surveying scenarios, the raw wavefront slope signals acquired by wavefront sensors are often non-stationary mixed signals. The noise mechanisms they contain are extremely complex, primarily resulting from the coupling of the following three physical components.

Intrinsic photonic noise: Due to quantum fluctuations in the laser source and the photoconversion process in the wavefront sensor array, the system inevitably contains shot noise and thermal noise. Although the energy at a single frequency point is low, this noise severely disrupts the geometric coherence of the optical signal at extremely low signal-to-noise ratios, resulting in extreme randomness and disorder in the optical phase in the high-frequency region of the complex spectrum.

Optical speckle and turbulent disturbances: When a coherent beam scatters off a target surface and passes through a dynamic, turbulent atmospheric medium, severe speckle effects and phase flickering occur. This spatially distributed random phase modulation manifests in the time domain as multiplicative distortion and additive crosstalk in the wavefront slope signal. Speckle noise exhibits highly nonlinear and nonstationary characteristics, causing the transient Doppler shift features of target vibrations to undergo severe broadening and blurring.

Environmental background noise: Under actual operating conditions, mechanical vibrations from the measurement platform itself and acoustic interference from the target’s surroundings introduce a significant amount of low-frequency, high-energy background Doppler shifts. The energy of this environmental background noise is often several orders of magnitude greater than that of the extremely faint vibrations of submillimeter-sized targets, thereby creating a strong “masking effect” in the time-frequency spectrum.

**Figure 5:**
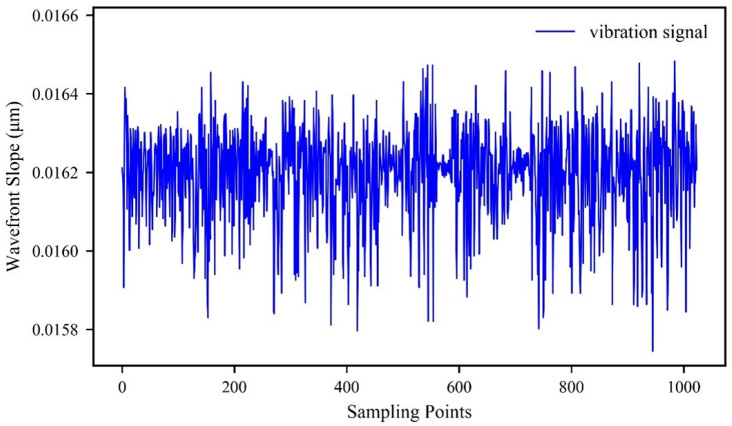
Noise waveform in an unshielded environment, affected by composite noise.

In real-world detection scenarios, the two types of physical interference mentioned above often couple and superimpose in both time and space, manifesting as highly random and complex composite background noise, as shown in Figure 5, which leads to a drastic deterioration in the fidelity of target echo signals. To address this hardware-level physical degradation, this study introduced an active optical isolation strategy during the signal acquisition phase: by fully encapsulating the wavefront sensor within a tightly sealed, light-shielded environment, external stray light paths and airflow interference are mitigated at the physical source, effectively ensuring the purity of the initial measurement data.

### 2.4. Creation of the Experimental Data Set

#### 2.4.1. Data Acquisition Equipment

**Figure 6:**
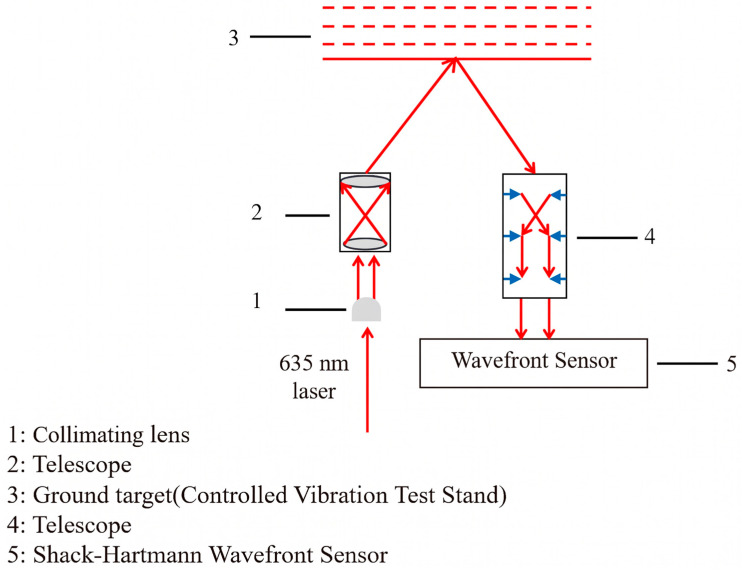
Complete experimental system.

Data acquisition utilized a 70-megawatt continuous-wave laser to simulate and detect longitudinal seismic vibration signals at a distance of approximately 10 m with high sensitivity; see Figure 6 for a detailed view of the complete experimental setup. To ensure a highly controlled detection environment and minimize interference from external factors such as stray light and air currents, the entire optical remote sensing system was integrated within a shielding enclosure, thereby effectively enhancing the system’s stability.

The wavefront sensor utilizes a Thorlabs Shack-Hartmann wavefront sensor (Model: WFS20-5C) as its core detection element, which is used to precisely quantify the wavefront distortion in the echo caused by micro-vibrations on the target surface. This sensor offers wavefront reconstruction accuracy as high as λ/30 (RMS@633 nm) and supports high-speed sampling for capturing transient seismic waveform characteristics. With its integrated automatic shutter control technology, the wavefront sensor can adaptively adjust the input optical power across a wide dynamic range, ensuring the system maintains exceptional detection sensitivity under varying wavelength conditions. Detailed technical specifications of the sensor are shown in Table 1.

To capture wavefront slope signals under various conditions, we used a six-degree-of-freedom controlled vibration test stand to regulate amplitude and frequency, thereby achieving an optimal simulation of the P-wave characteristics observed in natural seismic events. During data acquisition, the laser beam generated by the laser generator is focused by the telescope and directed toward the intermediate surface above the controlled vibration test bench. The reflected beam is then reflected by the intermediate surface, refocused by the telescope, and received by the wavefront sensor, thereby capturing changes in the wavefront slope signal of the vibration.

We used the LabVIEW software platform (LabVIEW 2020.0 (32-bit)) to monitor the response of the wavefront sensor in real time. To capture the transient changes caused by seismic waves, the system employed a high-speed sampling mode at 800 fps to obtain the wavefront slopes at the center of mass of the sensor’s 6 × 6 microlens array, and then reconstructed the corresponding wavefront image using the partitioning method.

#### 2.4.2. Wavefront Slope Signal Dataset

**Figure 7:**
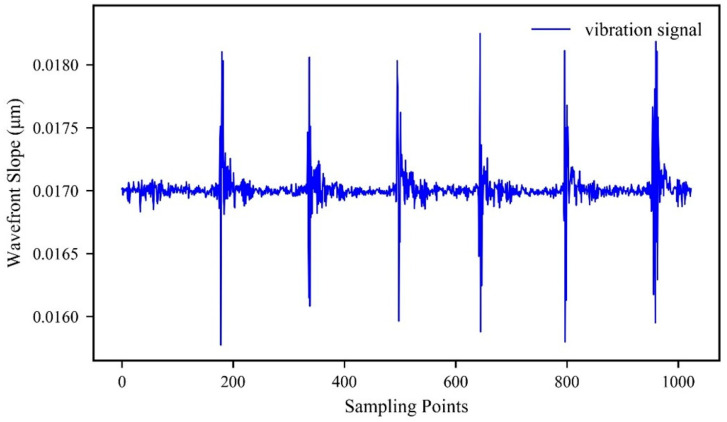
Clean wavefront slope signal acquired by the wavefront sensor using a mask. It can be seen that even the clean signal acquired under masked and nighttime conditions contains some background noise.

**Figure 8:**
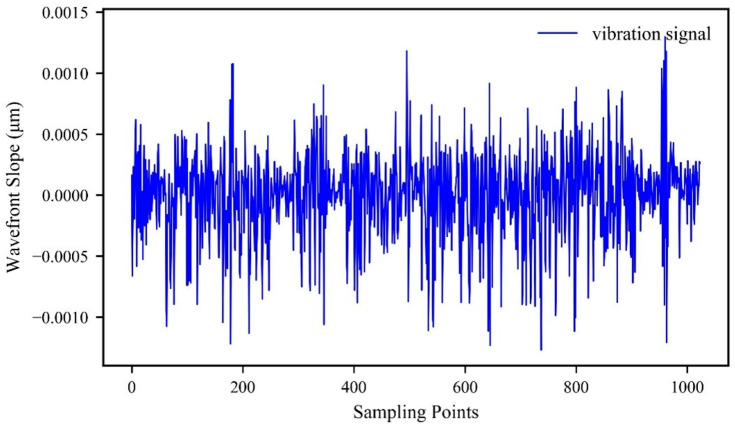
Wavefront slope signal acquired by the wavefront sensor without a mask; the signal is almost completely drowned out by various types of environmental noise.

Based on the large volume of data collected by the laser remote sensing seismic wave detection system, the dataset was divided into two categories based on whether a shield was used. Clean signals were acquired during nighttime hours, when interference from human activities and mechanical vibrations was minimized, using a physical shield to isolate external noise sources such as ambient airflow and stray light, as shown in Figure 7. Noisy signals were collected during the daytime without the use of a shielding hood and contained a large amount of various types of environmental noise disturbances, as shown in Figure 8.

Both the training set and validation set used in this study were obtained from the aforementioned data acquisition system in real-world environments, while the test set consists of data collected from real-world environments and synthetic data with varying signal-to-noise ratios (the synthetic test set with varying signal-to-noise ratios was created to verify the generalization capability of the PRISM network model under non-complex operating conditions). At the same time, we collected signals at different amplitudes (0.50, 0.62, 0.75, 0.81, 0.87, 0.93, 1.00, 1.12, and 1.18 mm) and discrete frequencies (0.1, 0.15, 0.18, and 0.2 Hz). A total of 52,338 clean signal samples and 8067 pure noise signal samples were randomly selected as the training set. Based on a test set of 8152 noisy signals collected in a laboratory environment, we also randomly selected 3 pure noise samples from the 1886 pure noise samples used to synthesize the test set and added them to the 3718 clean signals used to synthesize the test set. Thus, 11,214 noisy signals with varying signal-to-noise ratio (SNR) levels were generated based on the 3718 clean signals and 1886 pure noise signals. The use of a large number of noisy signals—both collected from real-world environments and synthesized across different SNR ranges—as the test set is sufficient to validate the generalization capability and robustness of this project. The composition of the datasets is shown in Table 2. Each dataset includes 1024 sample points.

We will use the above dataset for future research, so it is not currently available to the public. As described in detail above, we have provided an account of the data collection process and the configuration of the wavefront sensors.

## 3. PRISM Network Architecture

Traditional complex-valued neural networks (CVNNs) and time-frequency convolutional networks (such as the standard U-Net) perform well in conventional denoising tasks, but their direct application to two-dimensional complex-valued wavefront signals with extremely low signal-to-noise ratios reveals fundamental limitations. On the one hand, the algebraic cross-multiplication of the real and imaginary parts in standard complex convolution leads to “strong phase noise coupling” between channels due to intense random noise, thereby exponentially amplifying phase distortion. On the other hand, conventional single-stage architectures and loss functions are highly prone to “over-smoothing” of background noise, resulting in the permanent loss of fragile sub-millimeter-scale vibration features.

**Figure 9:**
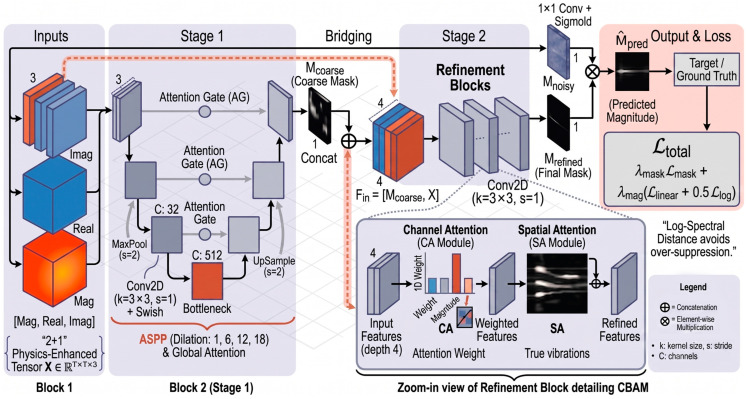
PRISM network architecture diagram.

Motivated by the above, this paper proposes a novel Physics-Aware Dual-Channel Decoupled Network (PRISM). As shown in Figure 9, this architecture primarily consists of four major components: physical tensor input, a dual-channel decoupling encoder-decoder, a cascaded refinement module integrating CBAM, and joint loss optimization. To completely break the cross-domain propagation paths of noise, its core dual-channel decoupling mechanism abandons standard complex calculations and instead treats the real and imaginary parts as mutually isolated, independent feature streams for parallel mapping. This not only enables the network to accurately capture phase continuity in an interference-free high-dimensional space but also requires only two real-number convolutions per operation, reducing theoretical computational overhead by nearly 50% at the fundamental level. Furthermore, regarding joint loss optimization, to address the issue of “over-suppression”—where a single Mean Squared Error (MSE) metric can cause the network to fall into a “zero-convergence” trap and thereby obscure subtle peaks—the model specifically introduces Log-Spectral Distance (LSD). Leveraging the high gradient sensitivity of the logarithmic domain, LSD non-linearly amplifies subtle feature errors, forcing the network to overcome global statistical inertia and thereby faithfully reconstruct minute transient physical harmonics that are otherwise drowned out by noise.

### 3.1. Overview of the Overall Architecture

Physical Enhancement Tensor Input. The system first converts a one-dimensional noisy wavefront slope signal into two-dimensional time-frequency features via a Short-Time Fourier Transform (STFT) [18]. To fully leverage the signal’s physical prior knowledge, the network constructs a physical enhancement tensor (PET) of size *T* × *F* × 3 as a unified input. This tensor explicitly separates the amplitude (Mag), real part (Real), and imaginary part (Imag) across the depth channels, laying the physical foundation for subsequent phase decoupling.

Stage 1: Coarse Phase Estimation. This stage serves as a preliminary filtering module, with its core mechanism involving the use of dual-channel decoupled convolutions to process real- and imaginary-part features separately, thereby physically blocking phase noise coupling caused by complex cross-terms. Through a bottleneck layer integrating ASPP (Atrous Spatial Pyramid Pooling) [19] and global self-attention, the network captures large-scale Doppler shift trends, ultimately outputting a coarse screening mask $M_{coarse}$ that has been preliminarily filtered to remove environmental background noise.

Feature Bridging and Stage 2 Refinement. To recover fragile vibration features that may have been lost during the initial filtering stage, the network performs channel-wise concatenation (bridging) between the original physical tensor and $M_{coarse}$. Stage 2 deeply integrates CBAM [20], where channel attention adaptively enhances the energy distribution, and spatial attention acts like a “microscope” to pinpoint the physical trajectories of genuine vibrations in the two-dimensional time-frequency domain. This ultimately outputs a high-fidelity refined mask $M_{refined}$ that preserves faint vibration signals amidst the noise while effectively filtering out a portion of the background noise.

Physical Reconstruction and Loss Optimization. In the final data flow stage, physical reconstruction is executed through the element-wise multiplication of the refined mask and the original noisy amplitude spectrum. This operation mathematically filters out the noise while retaining the exact energy distribution of the target signal. The entire network is trained end-to-end and constrained by a joint physical loss function that notably incorporates the Logarithmic Spectral Distance (LSD) [21,22]. The logarithmic nature of the LSD exponentially amplifies the penalty for distortions in micro-peaks, thereby eliminating the “over-killing” of weak signals often caused by standard Mean Squared Error (MSE) constraints. Finally, the system maps these high-fidelity time-frequency features back to the time domain via the inverse Short-Time Fourier Transform (iSTFT), ultimately outputting a clean, pure vibration waveform completely free of environmental interference.

### 3.2. Core Operator Configuration and Tensor Dimensional Evolution

**Figure 10:**
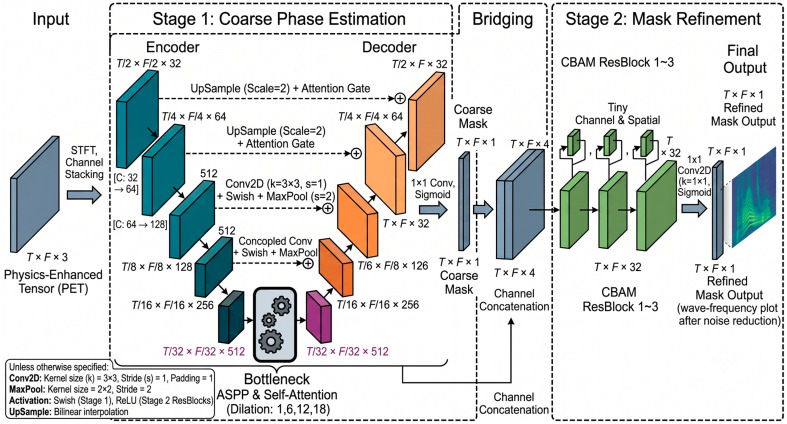
Configuration of core operators at each network layer and changes in the output tensor dimensions.

To clearly illustrate the intricate evolution of the feature space during the forward propagation process in the PRISM network, it is essential to map out the specific layer-by-layer transformations. Understanding this spatial and depth-wise transition is crucial for demonstrating the network’s efficiency, representational capacity, and structural logic. Figure 10 comprehensively presents the configuration of the core operators—including convolutional kernel sizes, strides, activation functions, and attention pooling mechanisms—at each distinct stage of the network. Furthermore, it explicitly tracks the corresponding changes in the dimensions of the output tensors as they transition from the high-resolution input space, compress into the deep semantic bottleneck, and are symmetrically upsampled back to the full-resolution masking space.

Encoder Evolution: The input tensor has dimensions *T* × *F* × 3. The encoder performs feature extraction through a five-layer progressive downsampling structure. The core operator of each layer consists of a decoupled convolutional layer (Decoupled Conv), the Swish activation function [23], and a max-pooling layer (MaxPool). The feature resolution is progressively downsampled from *T* × *F* to *T*/2, *T*/4, *T*/8, and *T*/16, until reaching *T*/32 at the bottleneck layer. Correspondingly, the channel depth gradually expands from 32 to 64, 128, and 256, ultimately reaching 512 at the bottleneck layer.

Bottleneck Layer Configuration: At the lowest resolution level of *T*/32 × *F*/32, a bottleneck block that integrates ASPP and self-attention mechanisms is deployed. The dilation rates of ASPP are set to (1, 6, 12, 18) to capture transient Doppler shift features at different scales.

Decoder and Mask Generation: The decoder restores spatial resolution through symmetric bilinear upsampling. Each layer performs feature fusion using attention gates and skip connections from the encoder, ultimately outputting a single-channel coarse-grained mask $M_{coarse}$ (*T* × *F* × 1) via convolution and a sigmoid activation function.

Stage 2: CBAM Refinement: Channel concatenation—after concatenating the coarse screening mask with the original physical tensor, the input dimension evolves to *T* × *F* × 4. Operator configuration: This stage consists of three cascaded CBAM residual blocks [24,25]. The data flow is first upsampled to a 32-dimensional feature space for fine-grained feature extraction, and is finally reduced to a single channel via convolution, outputting the final refined mask $M_{refined}$ (*T × F* × 1).

## 4. PRISM Noise Reduction Experiment and Analysis of Results

### 4.1. Experimental Parameter Settings and Evaluation Criteria

#### 4.1.1. Hardware Environment and Training Configuration

To ensure absolute fairness in experimental results and strict control of variables, all network training, internal ablation tests, and comparative evaluations of traditional baseline models in this study were uniformly conducted on high-performance computing servers equipped with NVIDIA A100 (40 GB VRAM) Tensor Core GPUs. This unified high-performance computing platform not only fully leverages the parallel processing advantages when handling large-scale, complex “2+1” dimensional physical tensors but also eliminates, at the physical hardware level, any interference from underlying computational fluctuations on model performance evaluations. Network model training was performed using Python 3.9 and TensorFlow 2.15.1 [26].

**Figure 11:**
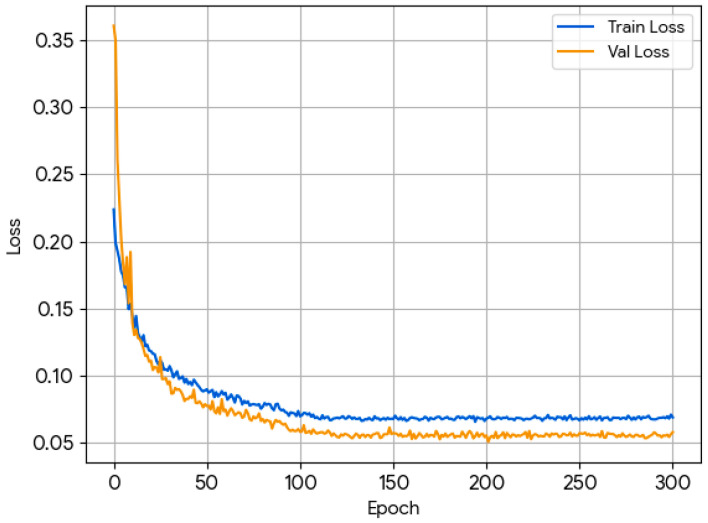
Training and validation loss curves over 300 epochs. It can be seen that the model’s convergence stabilizes after epoch 120.

The specific hyperparameter settings for the model training process are shown in Table 3. Figure 11 presents the convergence curves of the training and validation losses for the PRISM network. As shown in the figure, both the training loss and validation loss decrease steadily as the number of epochs increases. After 120 epochs, the two loss curves flatten out and no longer exhibit significant fluctuations, indicating that the model has converged and reached a stable state of fit.

In addition to the static initial hyperparameters specified in Table 3, this study specifically designs a dual-callback intervention mechanism within the training pipeline to enable dynamic tuning during the training process and prevent overfitting:

Adaptive learning rate decay: The algorithm monitors the validation set loss in real time. When the loss value plateaus for 15 consecutive epochs (no longer showing a downward trend), an annealing mechanism is triggered, which adaptively reduces the current learning rate by half to help the model converge more precisely around the optimal point.

Early Stopping Strategy: If the validation set loss shows no substantial improvement for 30 consecutive epochs, the system automatically determines that the model has reached its convergence limit and terminates training early. It then utilizes the model checkpoint mechanism to restore and save the model weights that performed best on the validation set, effectively preventing overfitting and wasting computational resources.

#### 4.1.2. Quantitative Evaluation Indicators

To comprehensively and objectively quantify the noise reduction performance and fidelity of the physical characteristics of the PRISM network under harsh conditions with extremely low signal-to-noise ratios (SNR), this paper overcomes the limitations of single-domain error analysis by establishing a six-dimensional evaluation system that encompasses time-domain morphology, frequency-domain energy, and overall distortion. The six core quantitative metrics selected are as follows:

Signal-to-Noise Ratio (SNR): Used to quantify the ratio of effective target energy to residual background noise energy in the denoised signal; it serves as a direct measure of the network’s pure “denoising” capability. Its calculation formula is:

(7) $$\mathrm{SNR}=10\log_{10}\left(\frac{\sum_{i=1}^{N}x{\left(i\right)}^{2}}{\sum_{i=1}^{N}{\left(x(i)-\hat{x}(i)\right)}^{2}}\right)$$

Here, *x*(*i*) represents the true, uncontaminated wavefront slope signal, and $\hat{x}(i)$ represents the denoised predicted signal output by the model. *N* is the total number of sampling points.

Signal Distortion Ratio (SDR): SDR is the most fundamental comprehensive metric for evaluating signal reconstruction quality [29]. As shown in Equation (8), where *s*(*i*) represents the original clean signal, $\hat{s}\left(i\right)$ represents the denoised signal output by the network, and *N* represents the number of sampling points. The numerator of the formula represents the energy of the original clean signal, while the denominator represents the reconstruction error energy (i.e., the sum of the residual background noise after denoising and the waveform distortion introduced by the algorithm). A higher SDR value indicates that the network has a stronger ability to suppress environmental noise and achieves better reconstruction fidelity.

(8) $$\mathrm{SDR}=10\log_{10}\left(\frac{\sum_{i=1}^{N}s^{2}(i)}{\sum_{i=1}^{N}(s(i)-\hat{s}(i){)}^{2}}\right)$$

Scale-Invariant Signal Distortion Ratio (SI-SDR): SI-SDR is the “gold standard” metric for evaluating the quality of deep learning-based signal reconstruction [30]. During wavefront slope signal reconstruction, the masking operations generated by the neural network may cause a slight gain deviation in the output signal relative to the original signal at the overall energy level. Traditional SDR penalizes this simple amplitude scaling as distortion, whereas SI-SDR introduces orthogonal projection techniques to project the reference signal *s* into the scale space of the predicted signal $\hat{s}$, thereby eliminating the interference caused by gain bias and purely evaluating the accuracy of waveform shape reconstruction. Its calculation formula is:

(9) $$\alpha =\frac{{\hat{s}}^{T}s}{\|s{\|}^{2}}$$

(10) $$s_{target}=\alpha s$$

(11) $$e_{res}=\hat{s}-s_{target}$$

(12) $$\mathrm{SI}\text{-}\mathrm{SDR}=10\log_{10}\left(\frac{\|s_{target}{\|}^{2}}{\|e_{res}{\|}^{2}}\right)$$

Here, *s* represents the original clean reference signal, $\hat{s}$ represents the predicted signal output by the network, α represents the scaling factor, $s_{target}$ represents the effective component in the estimated signal that is linearly correlated with the original signal, and $e_{res}$ represents the residual distortion component. In adaptive optics systems, the relative fluctuation trend of the slope signal (corresponding to the local phase of the wavefront) holds greater scientific value than its absolute physical amplitude; SI-SDR can more objectively reflect the PRISM network’s ability to capture the evolution patterns of wavefront phase.

Normalized Mean Squared Error (NMSE): Compared to the absolute mean squared error, NMSE eliminates evaluation biases caused by differences in signal amplitude by incorporating the original signal’s energy into the denominator, thereby focusing on evaluating the relative physical error of the reconstructed waveform. A lower NMSE indicates more accurate amplitude recovery of the waveform:

(13) $$\mathrm{NMSE}=10\log_{10}\left(\frac{\sum_{i=1}^{N}{\left(x(i)-\hat{x}(i)\right)}^{2}}{\sum_{i=1}^{N}x{\left(i\right)}^{2}}\right)$$

Pearson Correlation Coefficient (PCC): The PCC focuses on evaluating the consistency of the evolution of the “shape” of time-domain waveforms. It measures the degree of linear correlation between the denoised output and the clean signal, with values ranging from 0 to 1. The closer the PCC is to 1, the better the network preserves the phase information and the rhythmic fluctuations of the original weak harmonic waveform:

(14) $$\mathrm{PCC}=\frac{\sum_{i=1}^{N}(x(i)-\bar{x})(\hat{x}(i)-\bar{\hat{x}})}{\sqrt{\sum_{i=1}^{N}(x(i)-\bar{x}{)}^{2}}\sqrt{\sum_{i=1}^{N}(\hat{x}(i)-\bar{\hat{x}}{)}^{2}}}$$

(where $\bar{x}$ and $\bar{\hat{x}}$ represent the mean values of the true signal and the predicted signal, respectively). For an input signal containing noise, the values of SNR and SDR are identical because the interference in this case consists solely of additive random noise and contains no processing artifacts.

Log-Spectral Distance (LSD): Given that the wavefront slope signal contains subtle yet critical Doppler shift features, a single time-domain metric cannot capture the loss of high-frequency details present in the frequency domain. LSD is used to quantify the logarithmic difference between the amplitude spectra of the two signals in the frequency domain. This metric closely aligns with the Log-Spectral Mag Loss used during the PRISM training phase; a smaller LSD value indicates better spectral energy conservation by the model:

(15) $$\mathrm{LSD}=\sqrt{\frac{1}{K}\sum_{k=1}^{K}{\left(10\log_{10}|X(k)|-10\log_{10}|\hat{X}(k)|\right)}^{2}}$$

Here, |X(k)| and $\left|\hat{X}(k)\right|$ represent the spectral amplitudes of the clean signal and the reconstructed signal, respectively, in the *k*th frequency bin.

### 4.2. PRISM Performance Analysis

**Figure 12:**
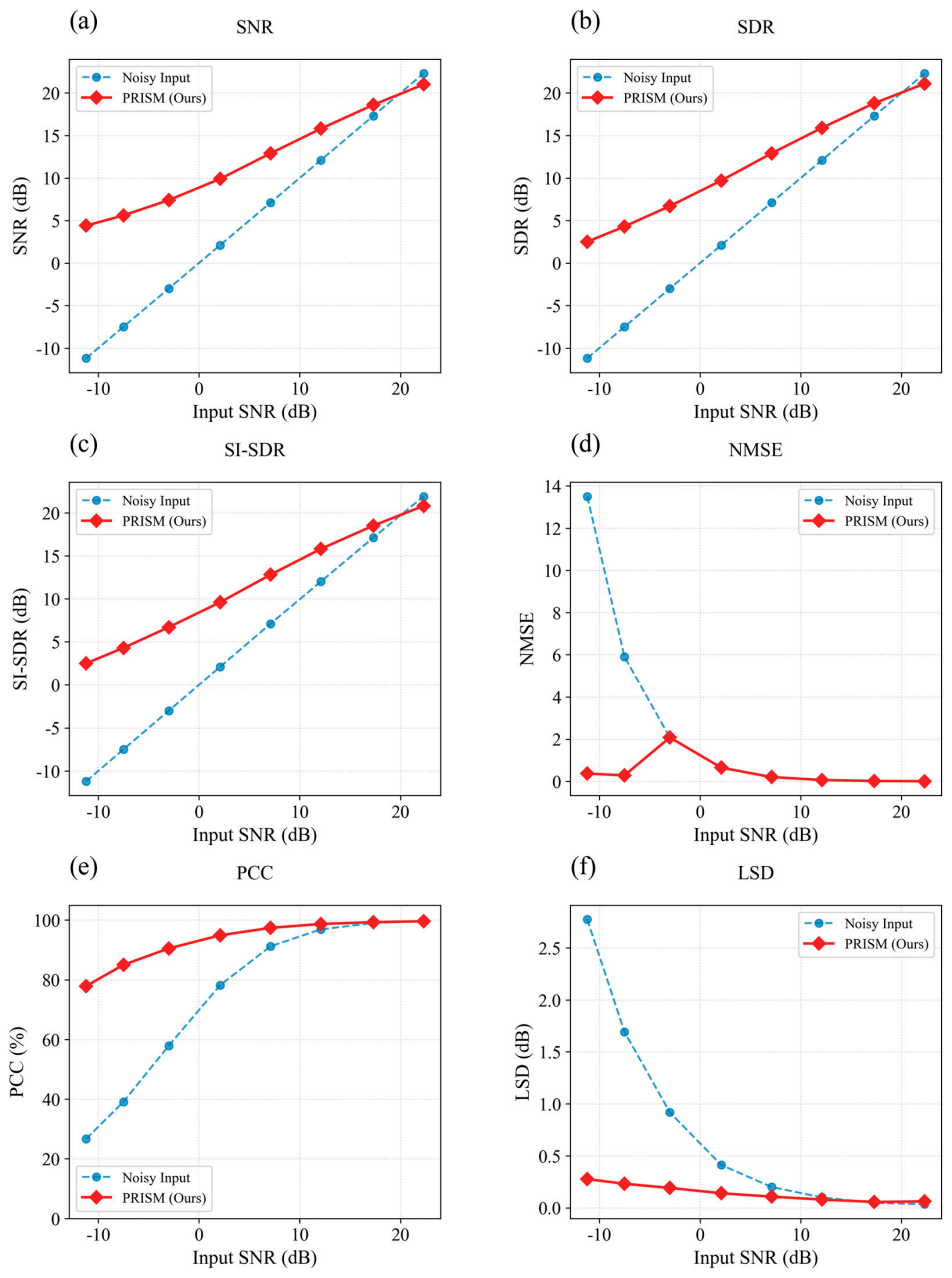
Line graph showing evaluation metrics for synthetic noise-containing signals with different signal-to-noise ratios.

As shown in Table 4, Table 5 and Table 6, the PRISM network architecture demonstrated strong performance across all noise reduction evaluation metrics when processing wavefront slope signals collected under laboratory conditions with multiple sets of excitation parameters (covering a range of sub-millimeter amplitudes and frequencies). As shown in Table 7, the model also achieved excellent macro-level average metrics across the entire test dataset. As shown in Figure 12, even when faced with complex, noise-contaminated signals generated artificially across multiple extreme SNR ranges, the model still achieved effective noise suppression and feature preservation. This cross-dataset validation fully demonstrates that the PRISM network not only possesses excellent robustness against interference but also exhibits strong generalization capabilities, thereby laying the technical groundwork for the future deployment of this model in complex and variable natural field environments or other advanced laboratory survey scenarios.

**Figure A1:**
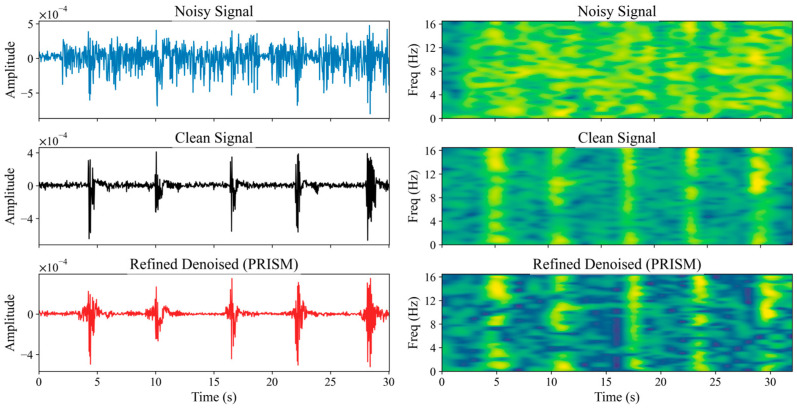
Time-frequency plot of a vibration signal with a frequency of 0.1 Hz and an amplitude of 0.50 mm.

**Figure A2:**
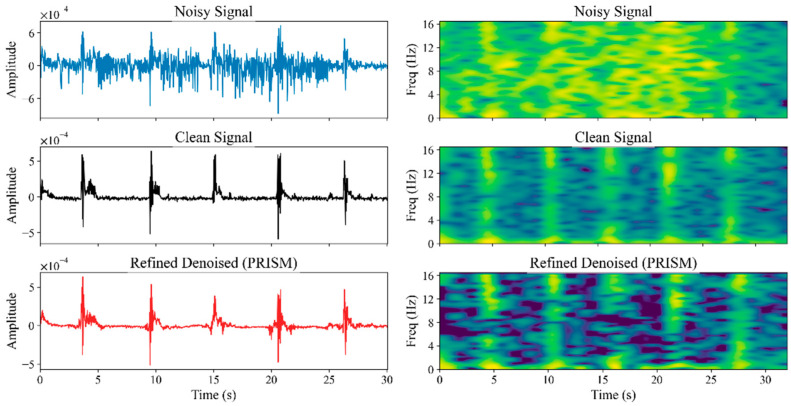
Time-frequency plot of a vibration signal waveform with a frequency of 0.1 Hz and an amplitude of 0.62 mm.

**Figure A3:**
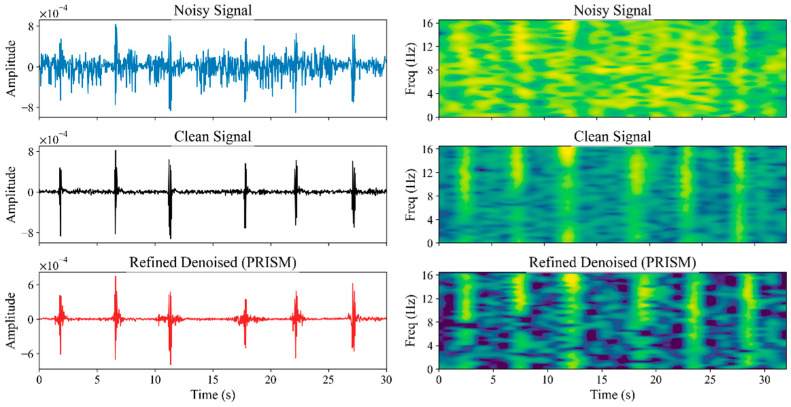
Time-frequency plot of a vibration signal waveform with a frequency of 0.1 Hz and an amplitude of 0.75 mm.

**Figure A4:**
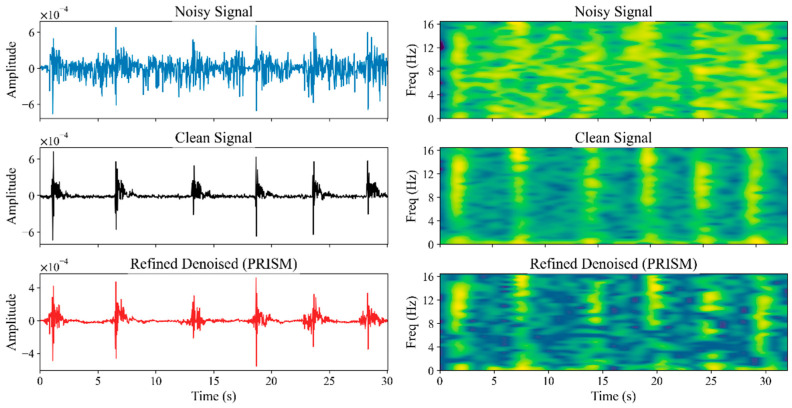
Time-frequency plot of a vibration signal waveform with a frequency of 0.1 Hz and an amplitude of 0.81 mm.

**Figure A5:**
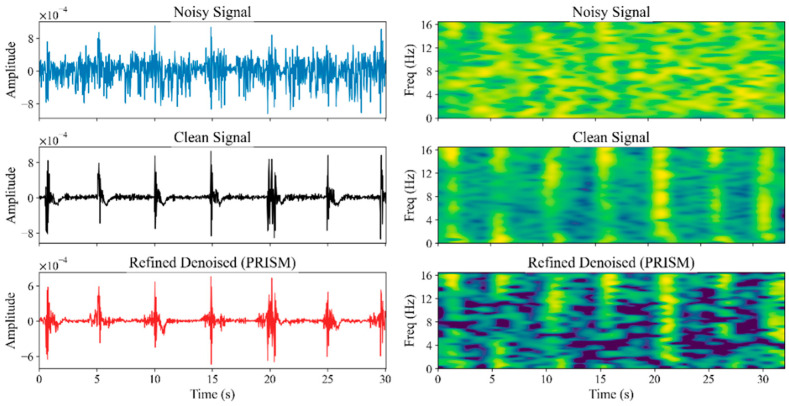
Time-frequency plot of a vibration signal waveform with a frequency of 0.1 Hz and an amplitude of 0.87 mm.

**Figure A6:**
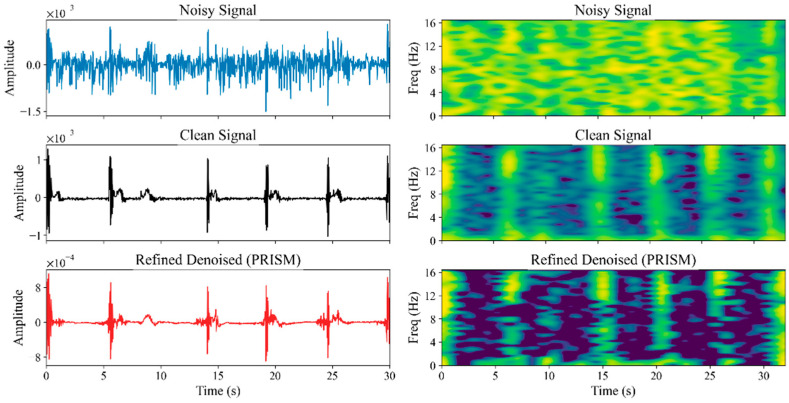
Time-frequency plot of a vibration signal waveform with a frequency of 0.1 Hz and an amplitude of 0.93 mm.

**Figure A7:**
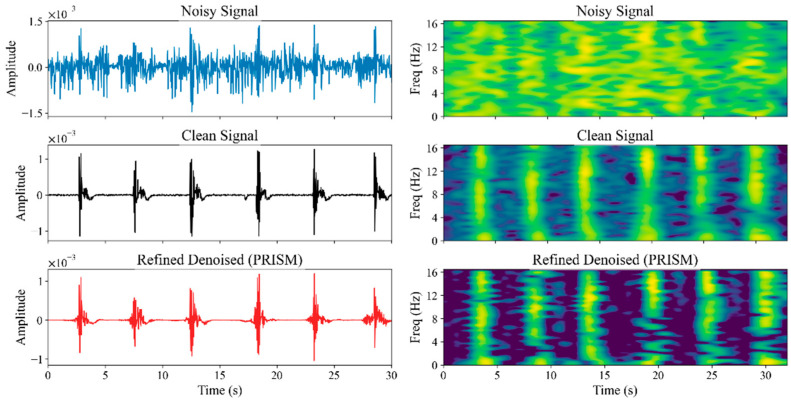
Time-frequency plot of a vibration signal waveform with a frequency of 0.1 Hz and an amplitude of 1.00 mm.

**Figure A8:**
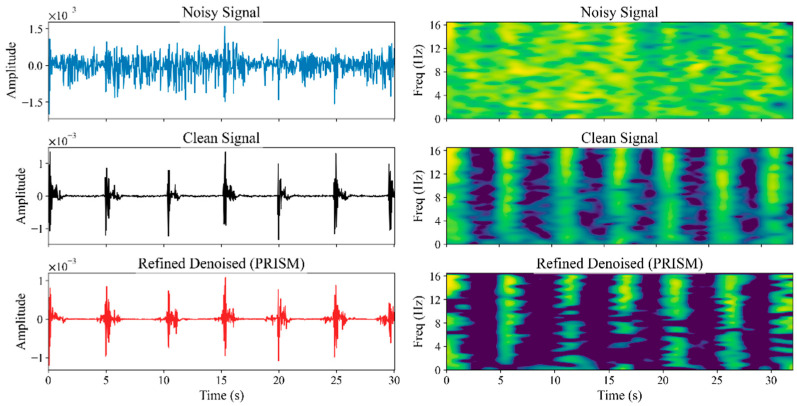
Time-frequency plot of a vibration signal waveform with a frequency of 0.1 Hz and an amplitude of 1.06 mm.

**Figure A9:**
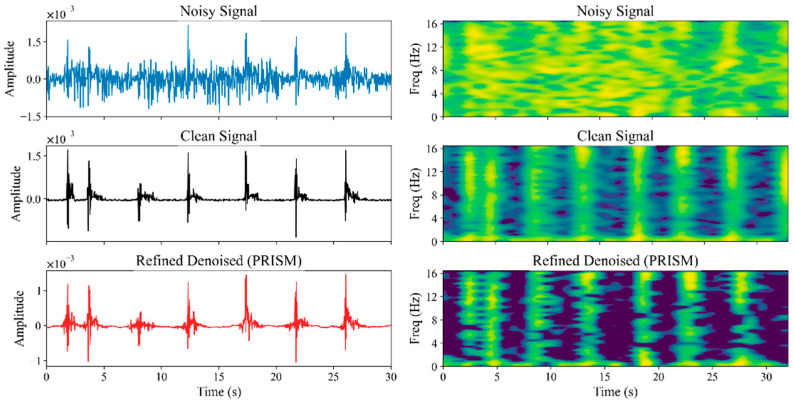
Time-frequency plot of a vibration signal waveform with a frequency of 0.1 Hz and an amplitude of 1.18 mm.

**Figure A10:**
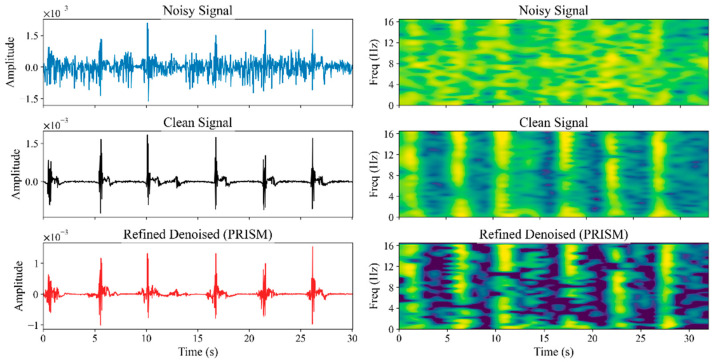
Time-frequency plot of a vibration signal waveform with a frequency of 0.1 Hz and an amplitude of 1.12 mm.

**Figure A11:**
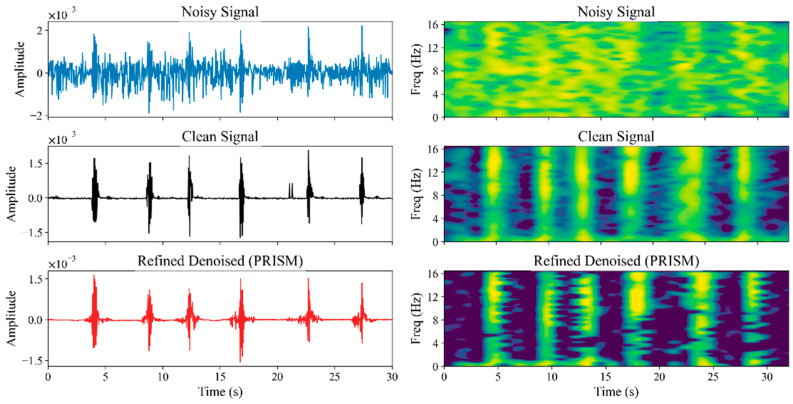
Time-frequency plot of a vibration signal with a frequency of 0.15 Hz and an amplitude of 1.06 mm.

**Figure A12:**
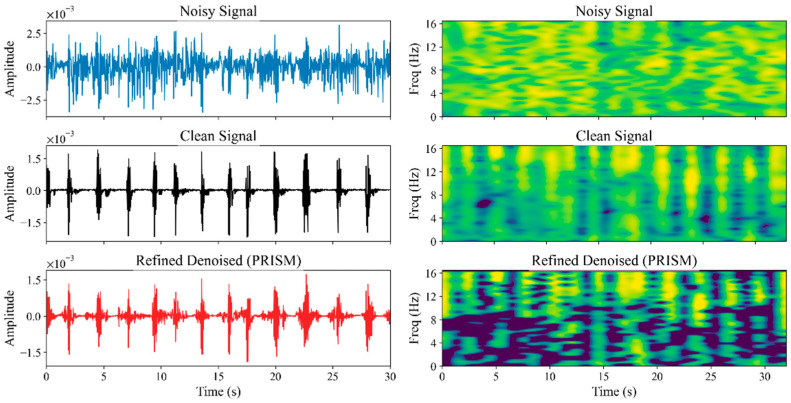
Time-frequency plot of a vibration signal with a frequency of 0.18 Hz and an amplitude of 1.06 mm.

**Figure A13:**
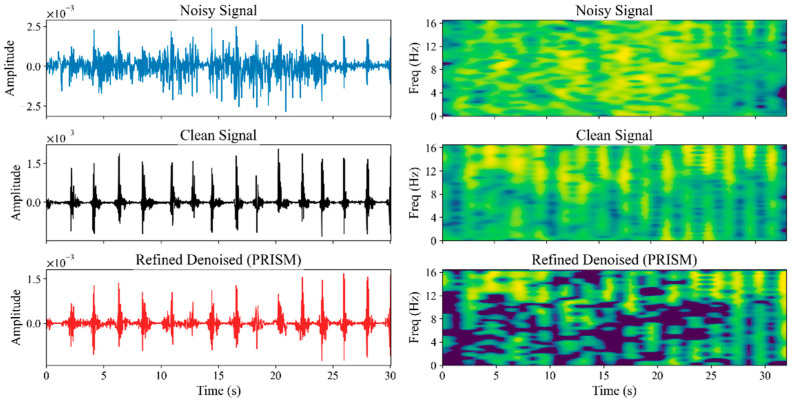
Time-frequency plot of a vibration signal with a frequency of 0.2 Hz and an amplitude of 1.06 mm.

It is worth noting that for signals that were originally relatively clean—i.e., test samples in high SNR ranges (such as [10,20])—the absolute improvement in some evaluation metrics was relatively limited, and in some cases, there were even slight fluctuations or declines (as shown in Figure 12). This is because the network model denoises some of the background noise in the originally relatively clean signals and better preserves the vibration signal features, which also indirectly demonstrates the advanced nature of the PRISM network model. As intuitively demonstrated by the time-frequency denoising results under different operating conditions in Appendix A (Figure A1, Figure A2, Figure A3, Figure A4, Figure A5, Figure A6, Figure A7, Figure A8, Figure A9, Figure A10, Figure A11, Figure A12 and Figure A13), the waveform baseline of the “Refined Denoised (PRISM)” signal processed by the network is even smoother and cleaner than that of the “Clean Signal” used as a reference benchmark, with significantly reduced background noise. This phenomenon not only explains the rationale behind the saturation of performance metrics at high signal-to-noise ratios but also, from the perspective of physically faithful reconstruction, profoundly validates the superiority of the PRISM network model in restoring the finest details.

In summary, the PRISM model demonstrated better noise reduction performance in tests using noisy signals collected in a real-world laboratory environment than in evaluations using synthetic noisy signals with varying signal-to-noise ratios. This indicates that the model possesses good generalization ability and robustness. To further clarify the effective boundaries and optimal application scenarios of the PRISM methodology, detailed data analysis indicates that in a real-world laboratory environment, the model achieves the highest reconstruction fidelity when the target microseismic signal frequency is 0.10 Hz (as shown in Table 5, with an SNR of 12.58 dB, with LSD as low as 0.1292 dB) and the amplitude is around 0.62 mm (as shown in Table 4, with PCC as high as 91.70%). This clearly indicates that PRISM demonstrates the most outstanding denoising performance in the low-frequency microseismic range of 0.10–0.20 Hz, particularly in the ultra-low-frequency band close to 0.10 Hz. Furthermore, the trend line of the synthetic signals (Figure 12) indicates that while the model achieved the best absolute evaluation metrics under extremely high input signal-to-noise ratios (>15 dB), its core strengths and greatest effectiveness are evident in the extremely low signal-to-noise ratio range (−11 dB to 0 dB). Within this range, the model successfully recovered severely distorted feature waveforms, achieving highly significant performance gains. This outstanding performance under various adverse conditions demonstrates that training with noisy signals across different signal-to-noise ratio ranges not only enables high-fidelity denoising of noisy signals in the laboratory environment described in this paper but also holds great potential for deploying and applying this model to feature extraction from complex noisy signals collected in natural environments or other laboratory settings. Figures illustrating the denoising results for noisy signals under different conditions are shown in Appendix A (Figure A1, Figure A2, Figure A3, Figure A4, Figure A5, Figure A6, Figure A7, Figure A8, Figure A9, Figure A10, Figure A11, Figure A12 and Figure A13).

### 4.3. Internal Data Flow Transformation and Visualization in PRISM

**Figure 13:**
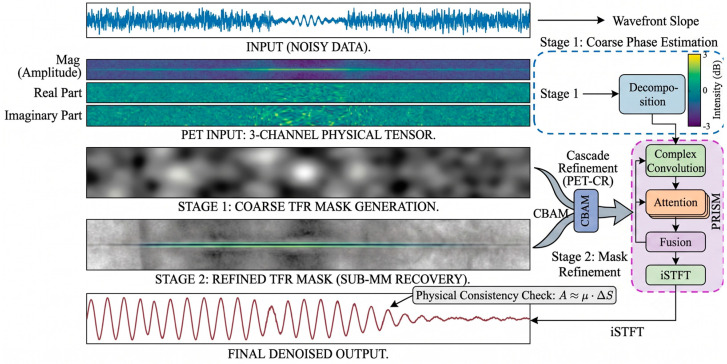
The evolution of the feature space for this sample across five key nodes within the network.

To thoroughly analyze the feature extraction mechanism of the PRISM network for weak wavefront slope signals and to validate the scientific validity of its “two-stage cascaded” design, this section provides a visual trace of the data flow within the network. Figure 13 illustrates the evolution of the feature space of a sample across five key nodes within the network.

#### 4.3.1. The Stepwise Evolution of Time-Frequency Features

**Raw Input and Physically Enhanced Tensor (INPUT & PET INPUT):** The system’s raw input (INPUT) consists of a one-dimensional, noisy wavefront slope signal with an extremely low SNR. At this stage, the time-domain waveform is chaotic, and the true vibration is completely obscured. After transformation, the signal is reconstructed as a three-channel “2+1”-dimensional Physically Enhanced Tensor (PET). As clearly shown in the figure, due to the deep coupling of turbulence and thermal noise, both the amplitude spectrum (Mag)—which represents energy distribution—and the real part (Real Part) and imaginary part (Imaginary Part)—which contain the phase evolution patterns of the wavefront core—exhibit highly random, fragmented background noise distributions. The faint Doppler shift trajectory of the target signal is difficult to discern amidst extremely strong interference.
**Stage 1: Coarse TFR Mask Generation:** The physical tensor enters Stage 1, where it undergoes decomposition to initially isolate phase cross-contamination, resulting in the generation of a coarse-resolution time-frequency mask for this stage. As shown in the figure, the feature map at this stage exhibits energy clusters with blurred edges and defocused, patchy patterns. This indicates that, while the macro-scale receptive field in Stage 1 successfully delineates the approximate frequency range of the target signal and strips away extensive peripheral background noise, it still lacks sufficient resolution for sub-millimeter-scale high-frequency transient details and fails to achieve precise energy convergence.
**Stage 2: Refined TFR Mask:** To achieve sub-millimeter-scale micro-reconstruction (Sub-mm Recovery), the coarse screening mask subsequently enters the Cascade Refinement (PET-CR) module, where it undergoes pixel-level calibration under the “microscope” magnification effect of the Convolutional Block Attention Module (CBAM). In this stage, the visual morphology of the feature space undergoes a significant qualitative transformation: through the joint optimization of complex convolution and attention modules, the originally defocused, blurry, and patchy energy clusters are highly compressed and reshaped into a highly contrasted, sharp, and continuous central horizontal bright line. This morphological reconstruction signifies that the network has successfully and precisely captured the true evolutionary trajectory of the target harmonic vibration.
**Final Denoised Output (FINAL DENOISED OUTPUT):** As the final physical feedback loop of feature evolution, the high-precision fine-grained mask is processed through feature fusion and inverse short-time Fourier transform (iSTFT), ultimately mapping back to a high-fidelity one-dimensional time-domain waveform (the red curve at the bottom). The reconstructed signal completely strips away the composite background noise and perfectly reproduces the target’s energy decay envelope and temporal coherence.

#### 4.3.2. Physical Consistency and Preservation of Form

When the reconstructed two-dimensional amplitude spectrum is mapped back to the one-dimensional time domain via the inverse short-time Fourier transform (iSTFT), the results are as follows:

Amplitude mapping accuracy: The final one-dimensional denoised waveform exhibits a high degree of consistency with the original clean signal in terms of amplitude magnitude, adhering to the physical mapping logic of A≈μ·ΔS. This demonstrates that the network possesses extremely high fidelity across the dynamic range.

Sub-millimeter-level fidelity: Even under extreme conditions with extremely low signal-to-noise ratios, PRISM successfully recovers subtle harmonic fluctuations without the phase drift or waveform clipping commonly observed in traditional methods.

The evolution of the aforementioned internal feature flow strongly demonstrates that the PRISM network is not merely a simple signal filtering tool, but rather achieves deep mining and precise reconstruction of weak physical features through real–imaginary decoupling and multi-stage refinement.

### 4.4. Comparative Experiments on Noise Reduction Performance

**Figure 14:**
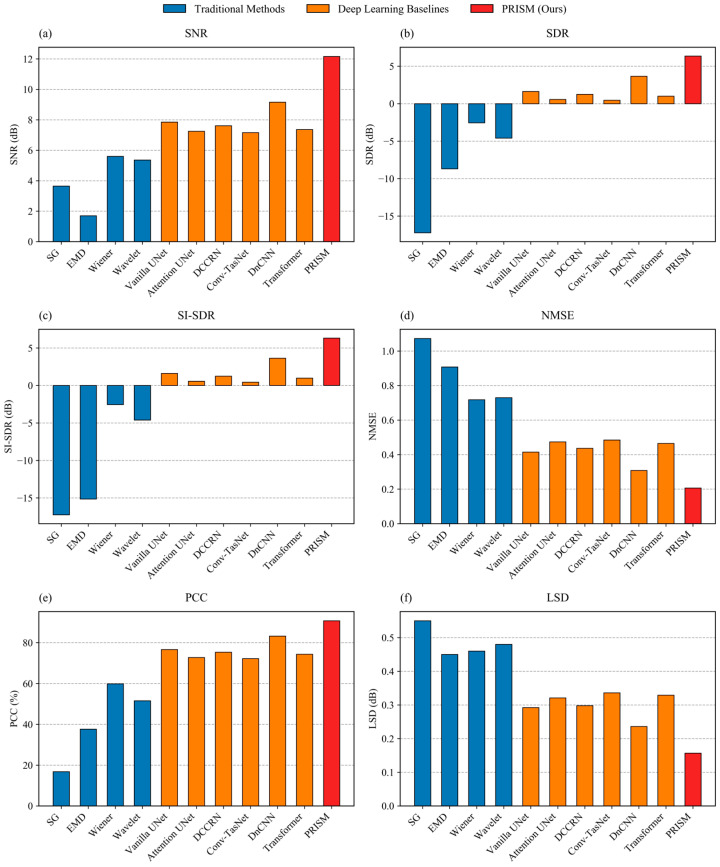
Performance of various metrics on noisy signals collected in a laboratory setting during comparative experiments.

**Figure 15:**
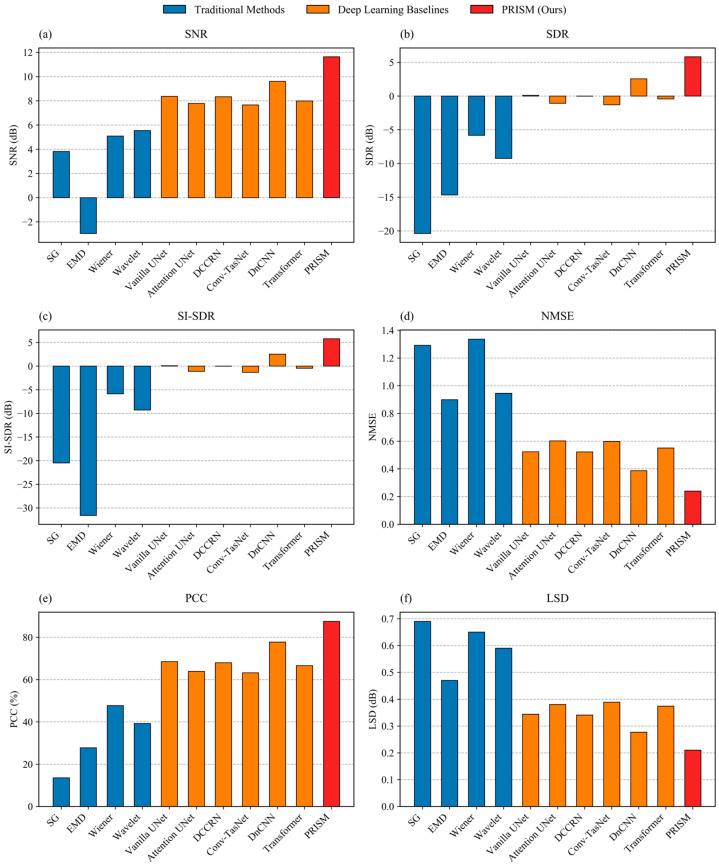
Comparison of the performance metrics of experimental models when synthesizing noisy signals with different signal-to-noise ratios.

To comprehensively evaluate the performance advantages of the PRISM model in the wavefront slope signal denoising task, this section presents a comprehensive comparison with two categories of mainstream baseline methods: one category consists of classic traditional signal processing algorithms [31,32,33,34] (including Wiener filtering, Savitzky-Golay (SG) filtering, wavelet transform, and empirical mode decomposition (EMD)); the other consists of representative state-of-the-art deep learning architectures [35,36,37,38,39] (including Vanilla UNet, Attention UNet, DnCNN, DCCRN, Conv-TasNet, and Transformer). The comparison metrics for experiments using noisy signals collected in a laboratory environment and synthetic noisy signals with varying signal-to-noise ratios are shown in Figure 14 and Figure 15.

As shown in Figure 14 and Figure 15, traditional signal processing methods (blue bars, such as SG, EMD, Wiener, and Wavelet) exhibit severe negative values for both SDR and SI-SDR metrics, and demonstrate high instability in both synthetic and real data. This indicates that traditional methods relying on fixed basis functions or linear threshold assumptions are unable to effectively decouple nonlinear phase distortions when faced with submillimeter-scale microseismic signals completely overwhelmed by strong background noise, resulting in irreversible and severe distortion of the reconstructed signal.

Among mainstream deep learning baselines (orange bar chart), although their overall performance surpasses that of traditional methods, specific metrics reveal critical flaws. For example, the SDR and SI-SDR of the Attention UNet and Vanilla UNet hover only around 0, and their NMSE is relatively high. This is because such conventional real-domain spatiotemporal networks heavily rely on macroscopic energy mapping, making them highly susceptible to the “oversmoothing” trap, where high-frequency transient harmonics at extremely low signal-to-noise ratios are mistakenly identified as random noise and filtered out. Furthermore, although DCCRN, specifically designed for complex signals, incorporates phase information, its metrics do not significantly outperform conventional networks and are even worse in some cases. This directly corroborates the theoretical inference presented earlier: under strong background interference, the algebraic multiplication of the real and imaginary parts in standard complex convolution causes severe “phase-noise strong coupling,” which not only fails to suppress noise but also exponentially amplifies the phase distortion of the target signal.

In contrast, the PRISM architecture proposed in this paper (red bar chart) demonstrates superior performance across all evaluation metrics in Figure 14 and Figure 15 (SNR exceeding 11 dB, PCC approaching 90%, and achieving the lowest NMSE and LSD), proving its exceptional generalization robustness between real and synthetic data. This performance is directly attributable to the physical rationality of its network design. The core dual-channel decoupling mechanism completely severs the noise propagation path between the real and imaginary parts caused by complex algebraic multiplication, ensuring that weak phase features can be extracted independently and with high fidelity even under extremely low signal-to-noise ratios. Simultaneously, the low LSD score indicates that the joint loss function successfully overcomes the global statistical inertia caused by the single mean squared error. Leveraging the high gradient sensitivity of the logarithmic domain, it prevents the network from over-suppressing weak features, thereby accurately restoring minute physical harmonic peaks.

These quantitative results fully demonstrate that, when handling complex and variable synthetic environmental noise, PRISM effectively balances the reconstruction of time-domain waveform morphology with the preservation of high-frequency spectral features, achieving leading performance in both the ultimate reconstruction accuracy of wavefront slope signals and overall generalization robustness.

### 4.5. Ablation Experiment

To investigate the contributions of each core innovative module in the PRISM network to the noise reduction performance of wavefront slope signals, this section presents a rigorous stepwise ablation study. The experiments begin with a baseline network stripped of all innovative modules, and then progressively introduce each mechanism to form four comparison models (M1 through M4). All models were trained and tested using the same hyperparameters and dataset. The module configurations for each model are shown in Table 8.

The overall performance of each model version across key quantitative evaluation metrics (i.e., SNR, SDR, SI-SDR, NMSE, PCC, and LSD) is shown in Table 9 and Table 10. A comparative analysis yields the following key conclusions:

By comparing the core quantitative metrics of each model, the effectiveness and necessity of the three major innovative modules in PRISM have been fully validated. After upgrading the baseline network M1 to the M2 model, which incorporates a “2+1” decoupled prior module, all evaluation metrics showed a stable upward trend across both test sets. This indicates that the decoupled prior mechanism can effectively separate the physical evolution patterns of the wavefront slope signal from the chaotic background noise, thereby providing a cleaner and more focused input foundation for the extraction of deep-layer features in subsequent networks. Furthermore, after introducing the CBAM cascaded refinement mechanism to form the M3 model, waveform fidelity metrics (such as PCC and SDR) achieved sustained improvements, while NMSE and LSD continued to decrease. This demonstrates that the channel and spatial attention mechanisms successfully guide the network to focus on high-value feature regions containing critical distortion information, effectively mitigating the issues of excessive smoothing and loss of high-frequency details commonly encountered in traditional denoising processes.

Building on this foundation, when the single MSE loss was replaced with a multi-metric joint loss function, the full-scale RISM network (M4) achieved the most significant performance leap. Taking the laboratory data in Table 9 as an example, the SNR metric showed a substantial improvement of nearly 1 dB (from 11.23 to 12.16), and the SI-SDR also exhibited a correspondingly significant enhancement. This result strongly confirms that, for physically complex signals such as wavefront slope, a single time-domain loss constraint is highly prone to trapping the model in local optima, whereas a multi-dimensional joint loss function can fully unleash the potential of the earlier network modules, guiding the model toward a global optimal solution.

It is particularly worth noting that, throughout the model evolution from M1 to M4, the evaluation metrics for both real-world laboratory datasets and synthetic datasets exhibited a perfectly consistent, gradually increasing trend. This strongly confirms that the three innovative modules in the PRISM architecture are not the result of mathematical overfitting to specific synthetic noise distributions, but rather have genuinely learned and mastered the universal physical laws governing the evolution of wavefront distortion signals. Each module is interlinked and indispensable, collectively endowing PRISM with exceptional noise reduction and fidelity preservation capabilities, as well as cross-domain engineering generalization potential.

## 5. Conclusions

To address the challenges of extremely low signal-to-noise ratios and phase coupling faced by Shack-Hartmann wavefront sensors when detecting weak seismic signals in extremely harsh environments, this paper proposes a novel Physics-Aware Dual-Channel Decoupled Network (PRISM). This study systematically explores the underlying physical mechanisms of wavefront slope signal denoising and achieves promising results.

PRISM deeply integrates three major innovative mechanisms. To address reconstruction artifacts caused by blind mapping in traditional complex networks, this paper innovatively proposes a “2+1” physical enhancement tensor and a dual-channel decoupling mechanism, successfully breaking the real-imaginary cross-interference in the complex feature space by utilizing amplitude priors. Concurrently, we constructed a “coarse-to-fine” cascaded network integrated with CBAM to precisely capture microscopic physical harmonics. Furthermore, by comprehensively introducing multi-dimensional physical joint constraints—including LSD—we effectively overcame the over-suppression of background noise caused by single-domain errors, achieving a global balance between waveform shape and energy conservation.

This paper established a dual-track validation loop combining “data collected from real-world laboratory environments” with “synthetic data at varying signal-to-noise ratios.” Extensive comparison and ablation experiments demonstrate that the PRISM architecture outperforms traditional signal processing methods and mainstream deep learning baselines in terms of generalization robustness and feature fidelity. This indicates significant potential for engineering generalization and practical application, providing a high-precision, highly generalizable universal solution for vibration signal denoising and extraction in extreme environments.

Although the PRISM model holds great potential for deployment in more complex natural field environments, providing robust data support for fields such as non-contact seismic exploration and deep geological structure inversion, this study still has certain limitations. The current architecture heavily relies on two-dimensional high-resolution time-frequency spectra as input features; this two-dimensional time-frequency masking estimation paradigm inevitably results in massive memory consumption and extremely high computational overhead. Consequently, achieving low-latency real-time deployment on portable wavefront sensor edge devices with strictly limited computational power remains a significant challenge. In future research, we will focus on exploring lightweight end-to-end networks that significantly reduce model parameters and computational complexity while maintaining the existing high-precision reconstruction capabilities, ultimately driving the evolution of intelligent wavefront sensing systems toward low-power, edge-side real-time computing.

## Figures and Tables

**Table 1:** Specifications of the WFS-20-5C Shack-Hartmann Wavefront Sensor.

Wavefront Accuracy	λ/30 (RMS@633 nm)
Microlens Pitch	150 μm
Microlens Focal Length	4.0 mm
Maximum Frame Rate	880 Hz (High-Speed Mode)

**Table 2:** Composition of the dataset.

	Clean Train	Noise Train	Real Noisy Test	Generate Noisy Test
Number	52,338	8067	8152	11,214

**Table 3:** Hyperparameter configuration.

Category	Parameter	Value/Setting
Input & Features	STFT window size (*N_win_*)	64
	Input feature shape	64 × 32 × 3
Network Structure	Base channels (*F_root_*)	64
	Encoder-decoder depth	5
Training Settings	Optimizer & Learning rate	Adam, 1 × 10^−3^ [27,28]
	Batch size & Epochs	512, 120
	Loss weights (*λ_mask_*, *λ_mag_*)	0.8, 0.2

**Table 4:** Evaluation metrics for vibration signals with a frequency of 0.1 Hz and amplitudes of 0.50, 0.62, 0.75, 0.81, 0.87, 0.93, 1.00, 1.06, 1.12, and 1.18 mm under laboratory conditions.

Evaluation Metrics in the Laboratory						
Amplitude (mm)	SNR (dB)	SDR (dB)	SI-SDR (dB)	NMSE (dB)	PCC (%)	LSD (dB)
0.50	12.14	6.31	6.26	0.2077	90.66	0.113
0.62	12.57	6.80	6.74	0.1903	91.70	0.106
0.75	12.21	6.41	6.37	0.2037	90.89	0.120
0.81	12.38	6.62	6.56	0.1969	91.32	0.112
0.87	12.42	6.73	6.70	0.1930	91.53	0.122
0.93	12.49	6.78	6.74	0.1914	91.65	0.095
1.00	12.52	6.77	6.76	0.1913	91.64	0.117
1.12	12.16	6.36	6.35	0.2056	90.86	0.129
1.18	12.25	6.53	6.50	0.1997	91.17	0.129

**Table 5:** Evaluation metrics under laboratory conditions for an amplitude of 1.06 mm across varying frequencies.

Evaluation Metrics in the Laboratory						
Frequency (Hz)	SNR (dB)	SDR (dB)	SI-SDR (dB)	NMSE (dB)	PCC (%)	LSD (dB)
0.10	12.58	6.72	6.69	0.1921	91.56	0.1292
0.15	12.23	6.38	6.34	0.2018	91.02	0.1485
0.18	12.01	6.21	6.19	0.2096	90.53	0.1651
0.20	11.82	6.09	6.06	0.2241	89.89	0.1852

**Table 6:** Average evaluation metrics for noisy signals collected in a laboratory setting.

Evaluation Metrics in the Laboratory						
	SNR (dB)	SDR (dB)	SI-SDR (dB)	NMSE (dB)	PCC (%)	LSD (dB)
Average	12.16	6.35	6.32	0.2069	90.75	0.157

**Table 7:** Average evaluation metrics for synthetic noise-containing signals with different SNRs.

Evaluation Metrics of Synthetic Different SNR Ratio Datasets						
	SNR (dB)	SDR (dB)	SI-SDR (dB)	NMSE (dB)	PCC (%)	LSD (dB)
Average	11.64	5.82	5.78	0.2395	87.51	0.21

**Table 8:** PRISM Ablation Experiment Configuration.

Model	Description	“2+1” Decoupled Prior	CBAM Cascade Refinement	Multi-Metric Joint Loss
M1	Vanilla Baseline	×	×	×
M2	Decoupled Model	√	×	×
M3	Cascade Model	√	√	×
**M4(Ours)**	**PRISM**	√	√	√

(Note: × indicates that the module is disabled and replaced with standard convolution or a single MSE loss; √ indicates that the innovative module is enabled).

**Table 9:** Evaluation metrics for ablation experiments using noisy signals collected in a laboratory environment.

Evaluation Metrics of Ablation Experiment (Laboratory)						
Model	SNR (dB)	SDR (dB)	SI-SDR (dB)	NMSE (dB)	PCC (%)	LSD (dB)
M1	10.54	5.00	4.98	0.2522	86.89	0.213
M2	10.87	5.28	5.26	0.2445	87.58	0.211
M3	11.23	5.53	5.51	0.2352	88.17	0.207
**M4(Ours)**	**12.16**	**6.35**	**6.32**	**0.2069**	**90.75**	**0.157**

**Table 10:** Evaluation metrics for ablation experiments using synthetic noisy signals with different signal-to-noise ratios.

Evaluation Metrics of Ablation Experiment (Different SNR)						
Model	SNR (dB)	SDR (dB)	SI-SDR (dB)	NMSE (dB)	PCC (%)	LSD (dB)
M1	10.52	4.16	4.13	0.3134	82.92	0.250
M2	10.66	4.49	4.49	0.3027	83.92	0.247
M3	10.85	4.81	4.77	0.2887	84.87	0.238
**M4(Ours)**	**11.64**	**5.82**	**5.78**	**0.2395**	**87.51**	**0.210**

## Data Availability

The data presented in this study are not publicly available due to ongoing future research utilizing this dataset. The data are available on reasonable request from the corresponding author.

## Abbreviations

The following abbreviations are used in this manuscript:

PRISM	Physics-Aware Dual-Channel Decoupled Network
CBAM	Convolutional Block Attention Module
ASPP	Atrous Spatial Pyramid Pooling
MLA	Micro-Lens Array
STFT	Short-Time Fourier Transform
iSTFT	Inverse Short-Time Fourier Transform
CMOS	Complementary Metal-Oxide-Semiconductor
PET	Physics-Enhanced Tensor
SNR	Signal-to-Noise Ratio
SDR	Signal Distortion Ratio
SI-SDR	Scale-Invariant Signal Distortion Ratio
NMSE	Normalized Mean Squared Error
PCC	Pearson Correlation Coefficient
LSD	Log-Spectral Distance

## Nomenclature

The following mathematical symbols and notations are used in this manuscript:

Physical and Optical Parameters	
Δz	Transient vertical vibration amplitude of the target surface
Δs	Spot centroid displacement on the CMOS focal plane
*f_ML_*	Intrinsic focal length of the microlens
α	Local tilt angle of the distorted wavefront
θ	Incidence angle of the probe laser beam
*M*	Lateral magnification factor of the receiving telescope
*R*	Wavefront radius of curvature at the micro-lens array
*K*	Intrinsic system conversion scale factor
Network and Signal Parameters	
*T*	Number of time frames (temporal dimension)
*F*	Number of frequency bins (spectral dimension)
*X*	“2+1”-dimensional Physics-Enhanced Tensor (PET)
$M_{coarse}$	Coarse-grained time-frequency mask generated in Stage 1
$M_{refined}$	High-fidelity refined mask generated in Stage 2
${\hat{M}}_{pred}$	Final predicted magnitude mask output by PRISM
x(i)	Original noisy wavefront slope signal
s(i)	Clean reference signal (Ground truth)
$\hat{s}(i)$	Denoised predicted signal reconstructed by the model
